# Body composition: a crucial factor in downstaging and postoperative complications of neoadjuvant chemotherapy for gastric cancer

**DOI:** 10.3389/fnut.2024.1481365

**Published:** 2024-11-20

**Authors:** Zhuanmei Jin, Min Chen, Qinglin Yang, Changyu Yao, Yanting Li, Taohua Zhang, Min Lai, Shuangxi Li, Lipeng Ding, Wenzhen Yuan

**Affiliations:** ^1^The First Clinical Medical College of Lanzhou University, Lanzhou, China; ^2^Department of Orthopedics, The First Hospital of Lanzhou University, Lanzhou, China

**Keywords:** gastric cancer, complications, surgery, descending phase, survival analysis, overall survival, body composition, nutritional status

## Abstract

**Background:**

Postoperative complications may lower the quality of life of patients, consequently leading to a reduction in their overall survival (OS). In our previous investigations, we found that patients with gastric cancer (GC) with postoperative complications who underwent direct surgery had a significantly lower OS than patients without complications. We observed no significant difference in OS among patients who underwent neoadjuvant chemotherapy (NAC), regardless of complications. We propose that for patients who underwent reoperation following NAC, downstaging (reduction of clinical stage) and postoperative complications exerted contrasting effects on the OS. Further, we hypothesize that post-NAC downstaging and the absence of postoperative complications lead to a longer OS.

**Methods:**

We conducted a retrospective analysis to collect the clinical data of patients with GC who underwent surgery after receiving NAC at the First Hospital of Lanzhou University from January 2016 to December 2022. Based on the presence of a post-NAC downstaging period and postoperative complications, we categorized the patients into group A (downstaging without complications), group B (downstaging with complications), group C (non-downstaging with complications), and group D (non-downstaging without complications). First, we assessed the OS disparity between the groups. Subsequently, we performed a comparative analysis of the body composition and hematological indexes of patients from the four groups.

**Results:**

We included 295 patients in the study and categorized them into four subgroups: group A comprised 83 patients (28.1%), group B comprised 32 patients (10.8%), group C comprised 83 patients (28.1%), and group D comprised 97 patients (32.9%). Group A patients had the longest OS of 40.1 ± 20.53, whereas group C patients had the shortest OS of 32.15 ± 25.09. The OS of patients in the other two groups was between these values. Pairwise comparisons revealed significant differences between the OS of group A patients and that of groups C (32.15 ± 25.09) and D (33.06 ± 20.89) patients (*p* < 0.05). The skeletal mass index (SMI) and skeletal mass area (SMA) were highest in group A, lowest in group C, higher in group A (SMI: 45.05 ± 7.44, SMA: 128.88 ± 22.67) than in group C (SMI: 41.61 ± 8.17, SMA: 115.56 ± 26.67) (*p* < 0.05), and higher in group D (SMI: 44.94 ± 6.87, SMA: 127.05 ± 23.09) than in group C (*p* < 0.05). However, we observed no significant difference between the SMI and SMA of groups B (SMI: 42.91 ± 9.68, SMA: 120.76 ± 30.51) and D (*p* > 0.05). With respect to hematological indexes, the prognostic nutritional index (PNI) was highest in group A and lowest in group C. The PNI in group A (417.89 ± 37.58) was significantly higher than that in group C (397.62 ± 47.56) (*p* < 0.05), and it was also higher in group D (410.76 ± 4.28) than in group C (*p* < 0.05). However, we observed no significant difference between the PNI in groups B (402.57 ± 53.14) and D (*p* > 0.05).

**Conclusion:**

Patients with advanced GC who experienced post-NAC downstaging and no postoperative complication had the longest OS. Patients with better body composition demonstrated more significant downstaging, fewer postoperative complications, and a longer OS.

## Introduction

Most patients with gastric cancer (GC) already have advanced-stage disease, which contributes to a poor overall prognosis (1). Reportedly, neoadjuvant chemotherapy (NAC) can enhance both the quality of life and survival rates of patients with advanced GC to a greater extent than surgery alone. This involved tumor downstaging, an increase in the complete resection rates, and elimination of potential micro metastases (2, 3). However, the effect of NAC on postoperative complications remains a topic of debate. In our previous investigations, we found that NAC is associated with an increased risk of postoperative complications, particularly grade 2 complications (see Appendix). Postoperative complications not only reduce the quality of life of patients with GC and prolong hospitalization but also exert a detrimental effect on overall survival (OS) and progression-free survival (4). However, some studies have indicated that postoperative complications in patients with GC who have undergone NAC surgery do not impact their OS (5). Our previous research has also confirmed the validity of this observation (see Supplementary material). To account for this interesting phenomenon, we posit that NAC introduces two confounding variables that affect the postoperative OS. On one hand, NAC increases the OS in certain patients by causing downstaging; on the other hand, surgical complications increase after NAC, leading to a reduced OS in some patients. Furthermore, we postulated that patients with advanced GC who exhibited a downstaging and experienced no postoperative complications after NAC demonstrated the longest OS. To test this hypothesis, we categorized patients who underwent surgery after NAC into four groups to investigate the impact of post-NAC downstaging and postoperative complications on OS in patients with advanced GC and elucidate the underlying mechanism.

## Method

### General information

We comprehensively reviewed the clinical, hematological, pathological, and imaging data of 295 patients with GC who underwent surgery after NAC at the First Hospital of Lanzhou University from January 2016 to December 2022. The inclusion criteria were as follows: (1) the presence of GC confirmed by preoperative histopathological biopsy results; (2) patients who underwent NAC treatment before a surgical procedure, NAC treatment includes SOX or SOX combined immunotherapy; (3) patients without contraindications to chemotherapy and radical surgery; (4) patients with comprehensive clinical data available. The exclusion criteria were as follows: (1) patients who received other treatments, such as interventional therapy or radiotherapy, before NAC or surgery; (2) patients with multi-organ dysfunction; (3) patients with other malignancies. Based on the presence of a post-NAC downstaging period and postoperative complications, we categorized the patients into group A (downstaging without postoperative complications), group B (downstaging with postoperative complications), group C (non-downstaging with postoperative complications), and group D (non-downstaging without postoperative complications).

### Postoperative complications

The occurrence of postoperative complications within 1 month was documented using the Clavien-Dindo classification system. These complications included bleeding, anastomotic fistula development, intestinal obstruction, incision infection, and lung infection, among others.

### Techniques used for acquiring and analyzing imaging data

**Figure 1:**
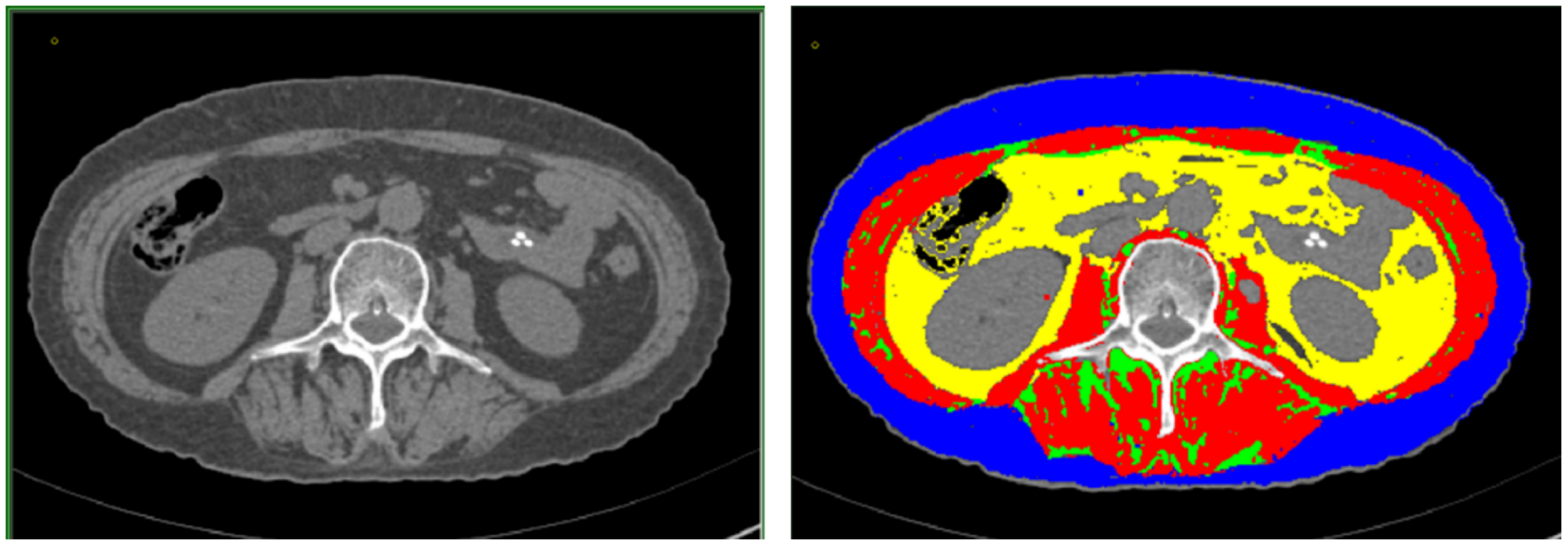
(Left) Cross-sectional image of the third lumbar vertebra acquired using computed tomography. (Right) sliceOmatic image of muscle fat composition at the third lumbar vertebra: visceral adipose tissue, intramuscular adipose tissue, subcutaneous adipose tissue, and skeletal muscle are marked by the yellow, green, blue, and red regions, respectively.

Computed tomography (CT) images of the third-last lumbar vertebra (L3) were acquired before NAC and before surgery (acquired at the same level based on feasibility) (Figure 1). According to the difference in the gray value of each body component in imaging [skeletal muscle (approximately −29 to +150 HU), subcutaneous fat (approximately −190 to −30 HU), visceral fat (approximately −150 to −50 HU), and intermuscular fat (approximately −190 to −30 HU)], we used the sliceOmatic software for the semi-automated analysis of skeletal muscle density (SMD), intermuscular fat density (IMFD), subcutaneous fat density (SFD), visceral fat density (VFD), skeletal muscle area (SMA), intermuscular fat area (IMFA), subcutaneous fat area (SFA), and visceral fat area (VFA). By calculating the ratio of each area to the square of height (cm^2^/m^2^), we sequentially calculated the skeletal muscle mass index (SMI), intermuscular fat index (IMATI), subcutaneous fat index (SATI), and visceral fat index (VATI).

### Formula for calculating hematological indices related to nutrition and inflammation

The serum test data hemoglobin (g/L), albumin (g/L), neutrophils (10^9^/L), lymphocytes (10^9^/L), monocytes (10^9^/L), and platelets (10^9^/L) were collected before NAC and before surgery. The neutrophile-to-lymphocyte ratio represents the ratio of the neutrophil count to the lymphocyte count. The systemic immunoinflammatory index is the ratio of the platelet count to the NLR. The platelet-to-lymphocyte ratio is the ratio of the platelet count to the lymphocyte count. The lymphocyte-to-monocyte ratio is the ratio of the lymphocyte count to the monocyte count. The prognostic nutritional index (PNI) is 10 times the albumin concentration (g/L) added to 0.005 times the lymphocyte count. SIINI is the product of the neutrophil count, platelet count, and hemoglobin level divided by the product of the lymphocyte count, body mass index (BMI), and albumin level.

### Statistical analysis

Data were analyzed using SPSS 26.0. The survival curves were prepared using GraphPad Prism 8.0. Measurement data are presented as ( $\bar{x}\pm s$ ), whereas counting data are expressed in percentage (%). We used the *χ*^2^ test, *t*-test, and *Z*-test to compare the relationships among classified, continuous, or hierarchical data. We used the *F*-test for group comparisons. We used the least significant difference method for pairwise group comparisons. We conducted Kaplan–Meier survival analysis to assess the impact of complications and downstaging on the OS. A statistically significant difference was indicated by *p* < 0.05.

### Patient survival follow-up

OS is the duration from the date of surgery to the date of death owing to any cause, or the duration from the date of surgery to the last follow-up, measured in months. We determined the patient’s survival status by consultation through the hospital information system and telephone. The most recent follow-up date was in January 2024.

## Result

### General patient characteristics

We enrolled 295 patients, including 231 males (78.3%) and 64 females (21.7%) (Table 1).

**Table 1:** General clinical data of patients.

Items	Types	*n*	%
Gender	Male	231	78.30%
	Female	64	21.70%
Age (in years)	<60	150	50.85%
	≥60	145	49.15%
Degree of differentiation	Medium and high	51	17.30%
	Poor	244	82.70%
Clinical stage	Stage II	50	16.95%
	Stage III	245	80.05%
Mode of operation	Laparotomy	223	75.60%
	Endoscopic	72	24.4%%
Body mass index	Malnutrition	21	7.12%
	Normal	129	43.73%
	Overweight	145	49.15%
Presence of postoperative complications	Yes	118	40%
	No	177	60%
Downstaging	No	184	62.40%
	Yes	111	37.60%

### OS

**Figure 2:**
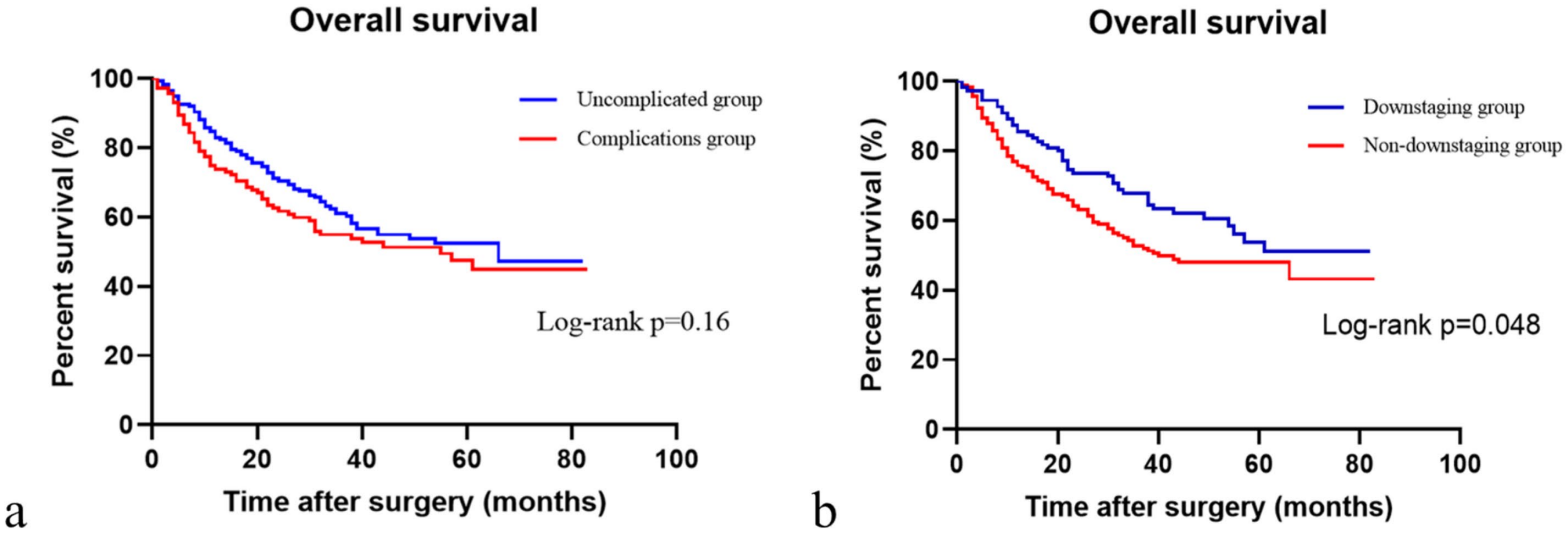
Comparison of overall survival with or without complications and downstaging or non-downstaging. **(a)** Comparison of overall survival in patients with or without complications. **(b)** Comparison of overall survival in the downstaging and non-downstaging groups.

We observed no significant difference in the OS between patients with and without complications following NAC and surgery (*p* > 0.05, Figure 2a). The OS of patients with post-NAC downstaging was significantly higher than that of patients without post-NAC downstaging (*p* < 0.05, Figure 2b).

### Subgroup analyses

**Figure 3:**
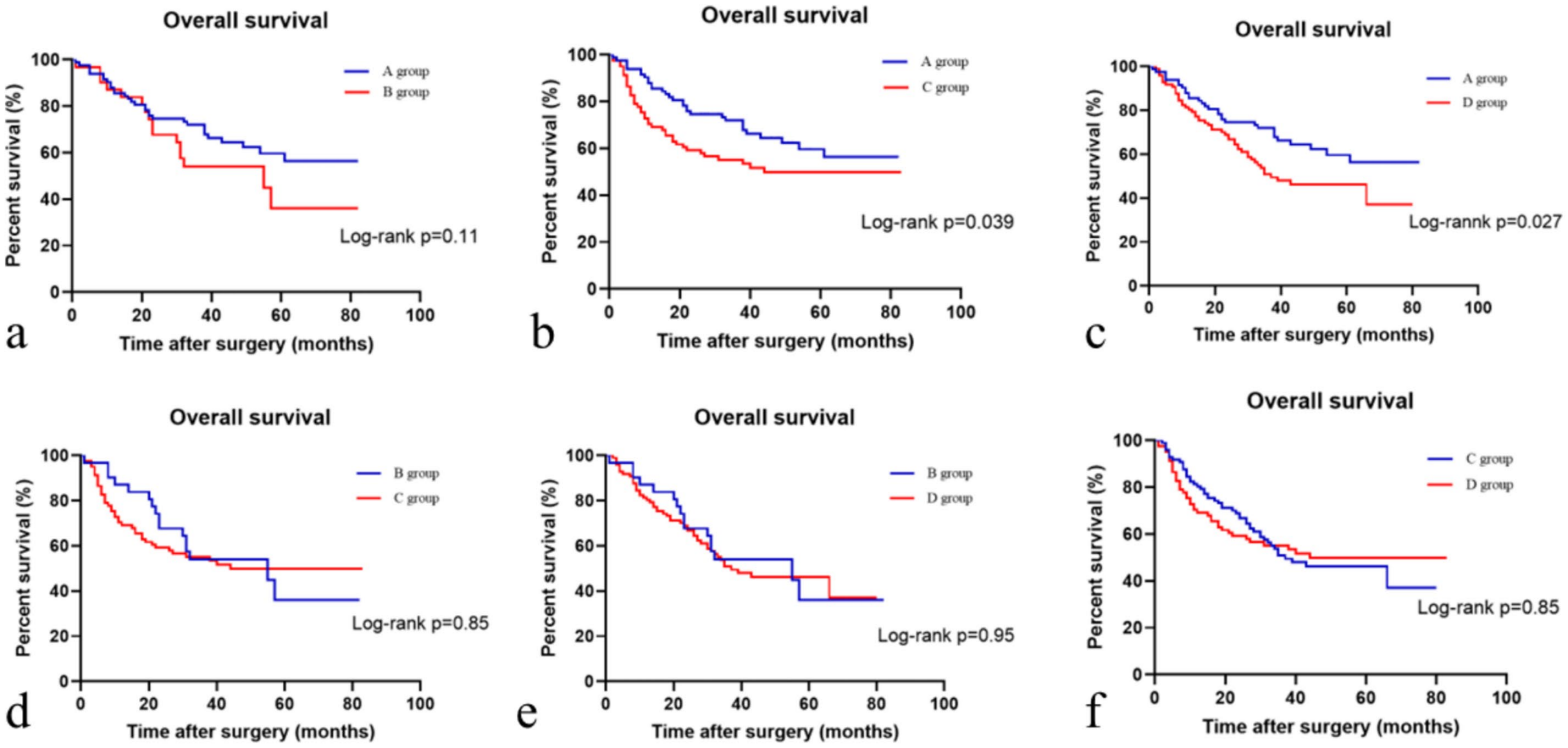
Comparison of overall survival in different patient subgroups. **(a)** Comparison of overall survival in the descending stage without complications and descending stage with complications groups; **(b)** Comparison of overall survival in the descending stage without complications and no descending stage with complications groups; **(c)** Comparison of overall survival in the descending stage without complications and no descending stage without complications groups; **(d)** Comparison of overall survival in the descending stage with complications and no descending stage with complications groups; **(e)** Comparison of overall survival in the descending stage with complications and no descending stage without complications groups; **(f)** Comparison of overall survival in the no descending stage with complications and no descending stage without complications groups.

Based on the presence or absence of post-NAC downstaging and postoperative complications, patients who underwent NAC surgery were divided into group A (downstaging without complications), group B (downstaging with complications), group C (downstaging with complications) and group D (non-downstaging without complications). Groups A, B, C, and D had 83, 32, 83, and 97 members, respectively. Group A patients had the longest OS (40.1 ± 20.53). Group C patients had the shortest OS (32.15 ± 25.09). Patients in the other two groups had intermediate OS values (Table 2). The OS of group A patients showed a statistically significant increase compared to that of group C and D patients, with respective *p* values of less than 0.05 (Figure 3).

**Table 2:** Fundamental data for each subgroup.

Group	*n*	%	Overall survival
Downstaging without complications (group A)	83	28.10%	40.1 ± 20.53
Downstaging with complications (group B)	32	10.80%	34.59 ± 19.66
Non-downstaging with complications group (group C)	83	28.10%	32.15 ± 25.09
Non-downstaging without complications group (group D)	97	32.90%	33.06 ± 20.89

### Body composition and hematological indicators

To investigate the factors contributing to the longer OS in group A patients, the shorter OS in group C patients, and the intermediate OS in the groups B and D patients, we assessed the body composition and hematological indicators of the participants. Among the various subgroups, group A patients had a significantly higher SMI (45.05 + 7.44) than group C patients (41.61 ± 8.17) (*p* < 0.05). Group C patients had a lower SMI (41.61 ± 8.17) than group D patients (44.94 ± 6.87) (*p* < 0.05). We observed no significant difference in the SMIs between group B (42.91 ± 9 0.68) and group D (44.94 ± 6.87) (*p* > 0.05, Table 3).

**Table 3:** Comparative analysis of the skeletal mass index of patients in various subgroups.

Group		*p*	95% confidence interval
Group A (83)	Group B (32)	0.18	[−1.03–5.32]
	Group C (83)	0.005	[1.07–5.81]
	D group (97)	0.93	[−2.17–2.39]
Group B (32)	Group C (83)	0.42	[−1.88–4.47]
	D group (97)	0.2	[−5.14–1.07]
Group C (83)	D group (97)	0.004	[1.05–5.61]

Comparison of the SMAs among subgroups revealed that group A patients had a significantly higher SMA (128.88 ± 22.67) than group C patients (115.56 ± 26.67) (*p* < 0.05). Additionally, group C patients had a lower SMA (115.56 ± 26.67) than group D patients (127.05 ± 23.09) (*p* < 0.05). The SMAs of group B patients (120.76 ± 30.51) and group D patients (127.05 ± 23.09) showed no significant difference (*p* > 0.05, Table 4).

**Table 4:** Comparative analysis of the skeletal muscle area of patients in various subgroups.

Group		*p*	95% confidence interval
Group A (83)	Group B (32)	0.12	[−2.19–18.2]
	Group C (83)	0.001	[5.59–20.82]
	Group D (97)	0.64	[−5.61–9.05]
Group B (32)	Group C (83)	0.32	[−4.99–15.40]
	Group D (97)	0.22	[−16.27–3.72]
Group C (83)	Group D (97)	0.002	[4.15–18.81]

Group A patients had a higher PNI (417.89 ± 37.58) than group C patients (397.62 ± 47.56), and group C patients had a lower PNI than group D patients (410.76 ± 4.28) (*p* < 0.05). The PNI of group B patients (402.57 ± 53.14) and group D patients (410.76 ± 4.28) showed no significant difference (*p* > 0.05, Table 5).

**Table 5:** Comparative analysis of PNI across patients in various subgroups.

Group		*p*	95% confidence interval
Group A (83)	Group B (32)	0.09	[−2.619–33.28]
	Group C (83)	0.003	[6.89–33.67]
	Group D (97)	0.28	[−5.76–20.04]
Group B (32)	Group C (83)	0.59	[−13.00–22.89]
	Group D (97)	0.36	[−25.78–9.39]
Group C (83)	Group D (97)	0.046	[0.24–26.03]

## Discussion

NAC has a dual impact. On one hand, it can lead to a reduction in tumor volume, TNM stage, and intraoperative spread and an increase in the R0 resection rate, thereby improving patient prognosis (6–8), consistent with the findings of this study. On the other hand, it may cause bone marrow suppression, reduce immune function, weaken the patient’s nutritional status and cardiopulmonary function, damage the intestinal barrier, and reduce the abundance of probiotic strains, besides causing other side effects (9–11). Furthermore, NAC may lead to local tumor tissue edema, exudation, and fibrosis. This potentially affects the delineation of anatomical gaps, increases the risk of intraoperative hemorrhage, prolongs the operative duration, and even compromises anastomotic healing. This eventually increases the likelihood of postoperative complications (12, 13). Previous research data suggest that post-NAC surgical intervention does not exhibit a significant association with OS in patients with GC, irrespective of the presence of complications or the occurrence of postoperative ≥II grade (14). We also identified this phenomenon in an earlier study. To further elucidate this phenomenon, we proposed a novel theory suggesting that patients with advanced GC who exhibit post-NAC downstaging without postoperative complications have the longest OS. Owing to the presence of two influencing factors (downstaging and postoperative complications), we divided the patients into four groups. Group A patients had the longest OS, whereas patients in group C had the shortest OS, with a statistically significant difference between the two groups. This finding validates our initial hypothesis. To further investigate why group A patients experienced significant downstaging and fewer postoperative complications, we assessed the body composition and nutritional status indicators of patients.

The term “body composition” refers to the contents of various physiological components (e.g., muscle, bone, fat, water, and minerals, among others), which indicate the proportional characteristics of the internal structure of the human body. Patients with cancer often suffer from disorders of skeletal muscle mass and fat consumption (15). Research findings indicate that the loss of muscle mass is associated with an increased risk of postoperative complications and is closely linked to a poor prognosis in patients with cancer (16). We found that patients with downstaging and without postoperative complications had the highest SMI and SMA, whereas patients without downstaging and with postoperative complications had the lowest SMI and SMA. Patients with downstaging and without postoperative complications had a significantly higher SMI and SMA than patients without downstaging and with postoperative complications (*p* < 0.05). The SMI and SMA of patients without downstaging or postoperative complications were higher than those of patients without downstaging with postoperative complications (*p* < 0.05). However, we did not observe significant differences in the SMI and SMA of patients between the downstaging with postoperative complications group and the non-downstaging without complications group (*p* > 0.05). This indicates that patients with better muscle mass have better downstaging, fewer complications, and a longer OS. Similarly, muscle loss is correlated with higher rates of postoperative complications and poorer long-term survival (17, 18). Patients with a better body composition experience fewer side effects of NAC, more significant downstaging, fewer surgical complications, and a longer OS (19, 20). We also found that the SMI and SMA of patients with complications were significantly lower than those of patients without complications. Additionally, the SATI, SFD, and VFA were higher in the downstaging group than in the non-downstaging group, whereas the changes in SMA, SFA, and SMI were lower in the non-downstaging group. The post-NAC changes in the SFA and albumin levels were negatively associated with the hemoglobin levels of patients, whereas changes in PNI and albumin were positively correlated. Furthermore, changes in SMI were negatively correlated with the post-NAC hemoglobin levels, whereas changes in the SMA were negatively correlated with the post-NAC hemoglobin levels (see Appendix). Therefore, variations in body composition may affect both downstaging and the occurrence of complications, thus exerting a significant influence on OS.

Skeletal muscle plays a central role in regulating glucose and insulin levels, facilitating fatty acid oxidation, and aiding amino acid storage. Adipose tissue plays a crucial role not only in maintaining insulin sensitivity and systemic balance but also in energy storage. Adipose tissue depletion often occurs faster and earlier than muscle loss, and the fat content can also affect the survival rates of patients with cancer (21, 22). Nevertheless, the effect of adipose tissue on survival remains a topic of debate, as some researchers propose that a high-fat mass is linked to cancer progression and poor post-surgery survival (23). Other researchers have indicated that patients with digestive tract cancer and low visceral fat content exhibit a shorter OS (24). Furthermore, among patients undergoing NAC treatment, a decrease in adipose tissue mass led to a shorter OS (25). This aligns with the results of this study, as we found that a higher fat content is positively correlated with survival. Hence, a higher skeletal muscle mass and both visceral and subcutaneous fat mass may be good prognostic factors for patients with GC undergoing surgery after NAC.

The preoperative nutritional status and postoperative complications play crucial roles in determining GC prognosis (26). Hemoglobin levels, PNI, and serum albumin levels are nutritional status indicators. Low hemoglobin levels before and after NAC indicate a compromised nutritional status as well as a high risk of anemia. Anemia increases the likelihood of postoperative infection, particularly pneumonia (27). Blood transfusion may ameliorate preoperative anemia; however, findings from several studies have indicated that this could potentially increase the recurrence rate and reduce the OS of patients with GC (28). Therefore, efforts should be made to prevent preoperative anemia in patients. The PNI, which is a nutritional prognostic index, is strongly associated with the levels of albumin and the lymphocyte count. Lymphocytes is not only the inflammation correlation index, in the process of anti-inflammatory, immune response and blood coagulation state plays an important role, is also a useful marker screening nutritional status (29). Lymphopenia lowers immunity and increases the risk of postoperative complications in patients. It can also suppress immune responses and cause cytotoxic damage-induced tumor progression (30, 31). A low preoperative PNI is correlated with the extent of tumor progression, which consequently reduces the survival rate of patients with GC (32). Consistent with our study findings, the PNI was higher in patients without complications and with downstaging, and their prognosis was better than that of other patients. Our findings revealed significant disparities in PNI across the four subgroups (*p* < 0.05), which prompted us to conduct pairwise comparisons of PNIs in the different groups. Group A had the highest PNI, whereas group C had the lowest, and the PNI in group A was significantly higher than that in group C (*p* < 0.05). Additionally, the PNI in group D was significantly higher than that in group C (*p* < 0.05), whereas the PNIs in groups B and D showed no significant difference (*p* > 0.05). These findings also explain why patients in group A had the longest OS, patients in group C had the shortest OS, and patients in groups B and D had intermediate OS. The results of our study also demonstrated that patients in the post-NAC downstaging group had a higher PNI, better nutritional status, fewer postoperative complications, and longer OS (see Appendix). This indicates that a higher PNI is associated with a better nutritional status, more obvious downstaging, fewer postoperative complications, and better OS.

Body composition and nutritional status are closely correlated. The deterioration of body composition can result in malnutrition and the weakening of immune function. Malnutrition can also lead to changes in body composition. However, body composition may play a more pivotal role. It may impact the tolerance toward OS after other treatments, such as surgery, by influencing changes in metabolism and inflammatory factors (33). Our findings showed that a better pre-NAC body composition was associated with more obvious downstaging, fewer toxic side effects from NAC, a better physical condition, fewer postoperative complications, and better survival. Hence, an optimal body composition, nutritional status, and energy reserve are essential for extending OS.

This study had both benefits and drawbacks. The benefits are as follows. We simultaneously investigated the effects of post-NAC downstaging and postoperative complications on OS. We evaluated the potential mechanism of action involved based on the body composition and hematological indexes of patients. The limitations were as follows. Since the study had a retrospective design, there may have been potential information bias. The lack of a standardized NAC regimen could lead to variations in the body composition, hematological indexes, nutritional indexes, and inflammatory indexes of patients before and after NAC. The sample size was relatively small because this was a single-center study. Lastly, we did not record progression-free survival data.

## Conclusion

Patients with advanced GC who underwent NAC and showed downstaging without experiencing postoperative complications had the longest OS. Patients with better body composition (indicated by SMI, SMA, and SFA) had better hematological parameters (such as PNI, hemoglobin, and albumin), fewer fluctuations, more significant downstaging, fewer surgical complications, and a longer OS after undergoing NAC. Based on our findings, we recommend clinicians to pay more attention to the physical status of patients. Furthermore, improving the nutritional status of patients and appropriately increasing pre-rehabilitation during NAC may help reduce hematological toxicity, improve downstaging, reduce complications, and prolong survival.

## Data Availability

The original contributions presented in the study are included in the article/Supplementary material, further inquiries can be directed to the corresponding author.

## Appendix

### Basic data of the two patient groups before and after propensity score matching

Before matching, group A comprised 295 individuals and group B comprised 151 individuals. The groups showed significant statistical disparities in the operation type (*p* < 0.05) and body mass index (BMI) (*p* < 0.05). The baseline data of the post-NAC surgery and the direct operation group were balanced and comparable at a 1:1 ratio through PSM. We used the operation type and BMI (BMI before NAC in the post-NAC surgery group; BMI before direct operation group before operation) for PSM. After PSM, baseline data were found to be balanced and comparable in both patient groups. This led to the successful matching of 266 patients (Supplementary Table S1). Based on the different treatment methods, patients in the post-NAC surgery were put into group A, and patients in the direct surgery group were put into group B.

### Clinical and pathological staging

To confirm the precision of clinical and pathological staging performed in this study, we compared the preoperative clinical stage and postoperative pathological stage as well as the pre-NAC clinical stage and preoperative clinical stage. We observed no significant difference between the preoperative clinical stage and postoperative pathological stage (*p* > 0.05, Supplementary Table S2). These findings suggest that the clinical staging results obtained in this study are valid. The pre-NAC clinical stage was higher than the preoperative clinical stage, with the difference being statistically significant (*p* < 0.05, Supplementary Table S3). This outcome demonstrates the efficacy of NAC in lowering the clinical stage.

### Comparison of overall survival in patients with or without complications before PSM

In group A, the OS of patients with and without postoperative complications showed no significant different (*p* > 0.05). However, in group B, the OS of patients with postoperative complications was significantly lower than that of patients without postoperative complications (*p* < 0.05, Supplementary Figure S1).

### Comparison of OS in patients with or without complications before and after PSM

In group A, patients with and without postoperative complications showed no significant difference in OS (*p* > 0.05). However, in group B, the OS of patients with postoperative complications was significantly lower than that of patients without postoperative complications (*p* < 0.05; Supplementary Figure S2 and Supplementary Table S4).

### Downstaging and non-downstaging groups

To assess the effect of post-NAC downstaging on OS and determine whether there are differences in body composition and hematological indicators between patients with post-NAC downstaging and no downstaging, we compared the groups separately.

With respect to body composition, the downstaging group showed higher SATI, SFD, and VFD than the non-downstaging group (*p* < 0.05). However, the downstaging group had lower ΔSMA, ΔSFA, and ΔSMI than the non-downstaging group (*p* < 0.05, Supplementary Table S5). Among hematological indices, the PNI in the downstaging group was significantly higher than that in the non-downstaging group (*p* < 0.05, Supplementary Table S6).

### Groups with and without complications

To assess the potential differences in body composition and hematological indicators between patients with and without postoperative complications, we compared the groups separately. Among body composition indicators, the SMI and SMA of patients without postoperative complications exceeded those of patients with postoperative complications (*p* < 0.05, Supplementary Table S7).

Among hematological indexes, the PNI, hemoglobin levels, NLR, and ΔSIINI of patients without postoperative complications were higher than those of patients with postoperative complications (*p* < 0.05). Conversely, the NLR, Δtriglyceride levels, SIINI, and ΔPNI of patients without postoperative complications were lower than those of patients with postoperative complications (*p* < 0.05, Supplementary Table S8).

### Four groups

We compared the indicators in the first four groups and subsequently evaluated the indicators that showed statistically significant differences pairwise between the groups. Group A had the highest SMI and SMA, whereas group C had the lowest SMI and SMA. Furthermore, we observed significant differences in the SMI and SMA among the four subgroups (*p* < 0.05, Supplementary Table S9). We also compared the hematological indexes among the four subgroups. Group A patients had the highest PNI, whereas group C patients had the lowest PNI. We observed significant differences among the PNIs of the four subgroups (*p* < 0.05, Supplementary Table S10).

### Correlation analysis

To investigate the correlation between changes in body composition before and after NAC in the downstaging group and hematological nutritional indices, we conducted Spearman correlation analysis using the change indexes of body composition before and after NAC. Correlation analysis revealed that the ΔSFA was negatively correlated with the post-NAC albumin and hemoglobin levels, whereas it was positively correlated with the ΔPNI and Δalbumin (Supplementary Table S11). We conducted a correlation analysis of ΔSMI and hematological nutritional indexes. ΔSMI was negatively correlated with the post-NAC hemoglobin levels (Supplementary Table S12). ΔSMA was also negatively correlated with the post-NAC hemoglobin levels (Supplementary Table S13). Supplementary Figure S3 shows the scatter plots depicting the ΔSFA, ΔSMA, and ΔSMI and hematological nutritional indexes.
