# Supplementary material for: Body composition: a crucial factor in downstaging and postoperative complications of neoadjuvant chemotherapy for gastric cancer
Source: Front Nutr. 2024 Nov 20;11:1481365. doi: 10.3389/fnut.2024.1481365 (PMC11614600; doi:10.3389/fnut.2024.1481365)
Supplement: Supplementary file 1 [file Data_Sheet_1.docx]

**Supplementary Material**

**
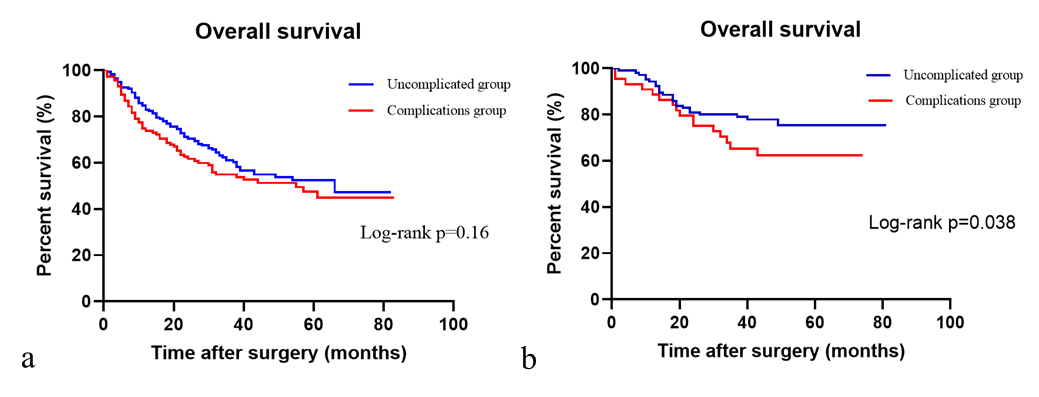
**

Figure 1. Comparison of the overall survival of patients with or without complications in the two groups before matching. (a) Group A, (b) group B.


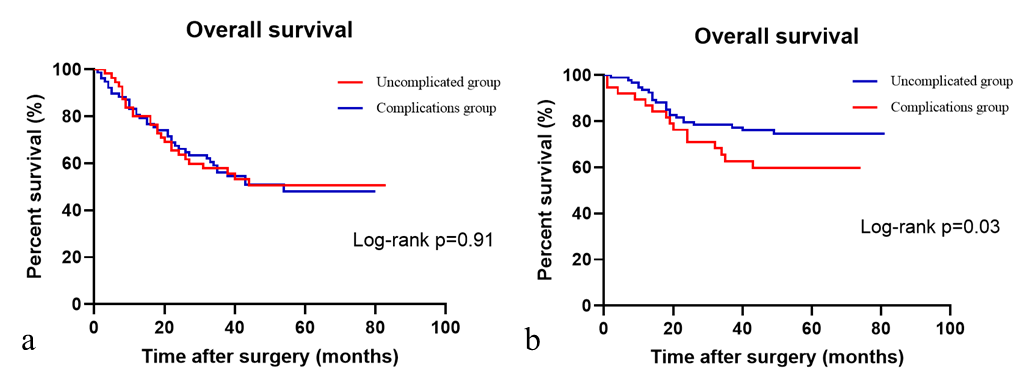


Figure 2．Comparison of the overall survival of patients with or without postoperative complications after the matching of the two groups. (a) Group A, (b) group B.

**
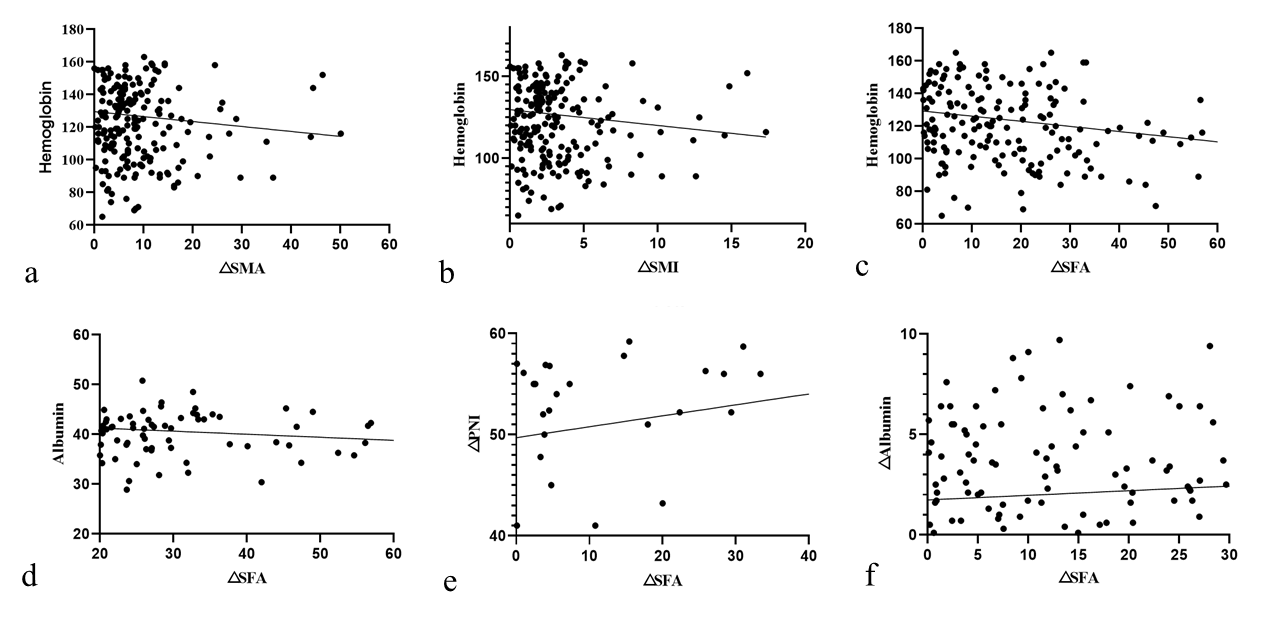
**

Figure 3. Scatter plot depicting correlations between body composition and hematological nutritional indexes before and after NAC. (a) △SMA and hemoglobin; (b) △SMI and hemoglobin; (c) △SFA and hemoglobin; (d) △SFA and albumin; (e)△SFA and △PNI; (f) △SFA and △albumin.

**Supplementary Material**

Table 1. Baseline data and propensity score matching results of the two groups

| Items | Types | Before propensity score matching | | χ2 | p | After propensity score matching | | χ2 | P |
| --- | --- | --- | --- | --- | --- | --- | --- | --- | --- |
|  |  | Group A (295) | Group B (151) |  |  | Group A (133) | Group B (133) |  |  |
| Gender | Male | 231 | 115 | 0.26 | 0.61 | 99 | 101 | 0.08 | 0.78 |
|  | Female | 64 | 87 |  |  | 34 | 32 |  |  |
| Age (in years) | <60 | 150 | 73 | 0.25 | 0.62 | 68 | 62 | 0.54 | 0.46 |
|  | ≥60 | 145 | 78 |  |  | 65 | 71 |  |  |
| Degree of differentiation | Medium and high | 51 | 36 | 2.73 | 0.1 | 23 | 29 | 0.86 | 0.35 |
|  | Poor | 244 | 115 |  |  | 110 | 104 |  |  |
| Clinical staging at initial diagnosis | Stage I | 0 | 39 | 105.1 | <0.01 | 0 | 32 | 46.3 | <0.01 |
|  | Stage II | 50 | 43 |  |  | 27 | 38 |  |  |
|  | Stage III | 245 | 68 |  |  | 106 | 62 |  |  |
|  | Stage Ⅳ | 0 | 1 |  |  | 0 | 1 |  |  |
| Body mass index | Malnutrition | 21 | 16 | 19.9 | <0.01 | 18 | 12 | 3.64 | 0.16 |
|  | Normal | 129 | 94 |  |  | 65 | 80 |  |  |
|  | Overweight | 145 | 41 |  |  | 50 | 41 |  |  |
| Mode of operation | Laparotomy | 223 | 85 | 17.4 | <0.01 | 83 | 84 | 0.02 | 0.89 |
|  | Endoscopic | 72 | 66 |  |  | 50 | 49 |  |  |
| Presence of postoperative complications | No | 177 | 105 | 3.91 | 0.05 | 77 | 93 | 4.17 | 0.04 |
|  | Yes | 118 | 46 |  |  | 56 | 40 |  |  |
| Downstaging | No | 184 |  |  |  |  |  |  |  |
|  | Yes | 111 |  |  |  |  |  |  |  |

| Table 2. Comparison of preoperative clinical stage and postoperative pathological stage in patients with gastric cancer | | | | | | | | |  |
| --- | --- | --- | --- | --- | --- | --- | --- | --- | --- |
| Group | Stage I | | Stage II | | Stage III | | Stage IV | |  |
| Preoperative clinical stage (295) | 35 | | 103 | | 157 | | 0 | |  |
| Postoperative pathological stage (295) | 50 | | 97 | | 147 | | 1 | |  |
| Z | -1.13 | |  | |  | |  | |  |
| p | 0.26 | |  | |  | |  | |  |
| Table 3. Comparison of pre-neoadjuvant chemotherapy clinical stage and preoperative clinical stage in patients with gastric cancer | | | | | | | | | |
| Group | | Stage I | | Stage II | | Stage III | | Stage IV | |
| Pre-neoadjuvant chemotherapy clinical stage (295) | | 0 | | 50 | | 245 | | 0 | |
| Preoperative clinical stage (295) | | 35 | | 103 | | 157 | | 0 | |
| Z | | -8.2 | |  | |  | |  | |
| p | | P<0.01 | |  | |  | |  | |

Table 4. Comparison of the overall survival of patients with and without postoperative complications before and after the matching of the two groups.

| Group (n) | Complications | No complications | χ2 | P |
| --- | --- | --- | --- | --- |
| Before-PSM patient (446) | 164 | 282 | 6.73 | 0.01 |
| Before-PSM Group A (295) | 118 | 177 | 1.99 | 0.16 |
| Before-PSM Group B (151) | 46 | 105 | 4 | 0.04 |
| After-PSM patient (266) | 96 | 170 | 3.36 | 0.07 |
| After-PSM Group A (133) | 56 | 77 | 0.01 | 0.91 |
| After-PSM Group B (133) | 40 | 93 | 4.55 | 0.03 |

| Table 5. Comparison of body composition between downstaging and non-downstaging groups | | | | | |
| --- | --- | --- | --- | --- | --- |
| Body composition indicators | Downstaging group (n=111) | Non-downstaging group (n=184) | t/z | p | 95% Confidence interval |
| SATI | 27.33±19.05 | 23.03±16.86 | 2.02 | 0.04 | [0.12-8.49] |
| IMATI | 2.59±2.43 | 2.19±1.6 | 1.7 | 0.09 | [-0.06-0.86] |
| VATI | 27.51±18.44 | 23.62±18.16 | 1.77 | 0.08 | [-0.43-8.21] |
| SMI | 44.59±8.2 | 43.34±7.62 | 1.32 | 0.19 | [-0.62-3.09] |
| SMD | 41.86±6.75 | 42.17±6.08 | -0.41 | 0.68 | [-1.81-1.18] |
| IMFD | -67.21±9.66 | -66.51±10.38 | -0.56 | 0.57 | [-3.08-1.71] |
| SFD | -84.41±13.26 | -80.81±12.82 | -2.3 | 0.02 | [-6.66--0.52] |
| VFD | -86.22±12.81 | -84.88±9.58 | -1.03 | 0.3 | [-3.93-1.23] |
| SMA | 126.76±25.46 | 121.71±25.22 | 1.66 | 0.1 | [-0.94-11.03] |
| IMFA | 7.33±6.65 | 6.15±4.53 | 1.82 | 0.07 | [-0.09-2.47] |
| SFA | 81.68±54.04 | 69.94±48.18 | 1.94 | 0.05 | [-0.19-23.61] |
| VFA | 74.65±53.83 | 60.88±48.56 | 2.27 | 0.02 | [1.81-25.74] |
| △SMA | 2.96±9.97 | 5.63±10.27 | -2.18 | 0.03 | [-0.56--2.63] |
| △SFA | 0.33±21.46 | 5.81±20.29 | -2.2 | 0.02 | [-10.39--5.77] |
| △VFA | -0.69±34.48 | 2.39±27.11 | -1.78 | 0.08 | [-5.56-0.27] |
| △IMFA | 6.42±12.26 | 7.32±12.46 | -0.60 | 0.55 | [-3.82-2.04] |
| △SMD | 0.78±3.55 | 0.99±4.16 | -0.45 | 0.65 | [-1.15-0.72] |
| △SAD | -0.35±9.31 | -2.48±9.56 | 1.88 | 0.06 | [-0.11-4.37] |
| △VAD | -3.26±12.08 | -2.31±8.05 | -0.82 | 0.41 | [1.17-1.34] |
| △IMFD | 2.41±10.72 | 0.03±10.02 | 1.93 | 0.05 | [-0.05-4.82] |
| △SMI | 1.01±3.45 | 2.05±3.67 | -2.41 | 0.02 | [-1.89--0l.19] |
| △IMATI | -0.22±2.22 | 0.16±1.22 | -2.01 | 0.05 | [-0.77--0.01] |
| △SATI | 1.65±12.06 | 4.33±13.29 | -1.69 | 0.09 | [-5.81-0.44] |
| △VATI | -1.67±14.31 | -1.26±13.67 | -0.24 | 0.8 | [-3.69-2.88] |

SATI, subcutaneous fat index; IMATI, intermuscular fat index; VATI, visceral fat index; SMI, skeletal muscle mass index; SMD, skeletal muscle density; IMFD, intermuscular fat density; SFD, subcutaneous fat density; VFD, visceral fat density; SMA, skeletal muscle area; IMFA, intermuscular fat area; SFA, subcutaneous fat area; VFA, visceral fat area; △, the change before neoadjuvant chemotherapy minus the change before operation.

Table 6. Comparison of hematological indicators between the downstaging and non-downstaging groups

| Various indicators | Downstaging group (n=111) | Non-downstaging group (n=184) | t/z | p | 95% Confidence interval |
| --- | --- | --- | --- | --- | --- |
| NLR | 2.41±2.58 | 2.64±2.97 | -0.72 | 0.48 | [-0.91-0.43] |
| SII | 239.89±410.32 | 190.59±215.48 | 1.35 | 0.18 | [-22.53-121.11] |
| PLR | 109.05±76.57 | 117.94±101.87 | -0.79 | 0.43 | [-30.93-13.15] |
| LMR | 3.87±1.59 | 4.86±17.96 | -0.58 | 0.56 | [-4.36-2.38] |
| PNI | 415.24±41.72 | 403.93±45.42 | 2.14 | 0.03 | [0.89-21.73] |
| SIINI | 44.17±62.78 | 53.29±87.43 | -0.96 | 0.34 | [-26.41-8.15] |
| Hemoglobin | 126.09±22.19 | 121.26±22.87 | 1.78 | 0.08 | [-0.52-10.18] |
| Albumin | 41.52±4.17 | 42.68±31.78 | -0.38 | 0.7 | [-7.12-4.81] |
| Platelet | 133.78±64.84 | 135.3±66.23 | -0.19 | 0.85 | [-17.08-14.00] |
| Cholinesterase | 5.85±1.2 | 5.87±5.1 | -0.06 | 0.96 | [-0.99-0.94] |
| Triglyceride | 1.39±0.71 | 1.38±0.65 | 0.13 | 0.89 | [-0.14-0.17] |
| High-density lipoprotein | 1.21±0.33 | 1.19±0.31 | 0.31 | 0.75 | [-0.06-0.09] |
| △NLR | 0.31±2.66 | 0.37±3.48 | -0.14 | 0.89 | [-0.81-0.71] |
| △SII | 181.29±608.38 | 216.46±532.17 | -0.52 | 0.6 | [-168.09-97.76] |
| △PLR | 56.2±97.01 | 65.97±165.22 | -0.57 | 0.57 | [-43.71-24.16] |
| △LMR | 2.81±20.82 | 0.49±23.11 | 0.87 | 0.39 | [-2.94-7.59] |
| △PNI | 16.85±42.89 | 19.16±56.35 | -0.37 | 0.71 | [-14.54-9.91] |
| △SIINI | 34.21±70.16 | 38.79±116.53 | -0.38 | 0.7 | [-28.63-19.44] |
| △Albumin | 1.68±4.29 | 1.91±5.65 | -0.37 | 0.71 | [-1.46-0.99] |
| △Hemoglobin | 11.13±17.73 | 8.15±22.06 | 1.21 | 0.23 | [-1.87-7.85] |
| △Platelet | 89.53±81.44 | 93.61±87.64 | -0.39 | 0.69 | [-24.27-16.11] |
| △Cholinesterase | 0.97±1.23 | 1.22±8.83 | -0.29 | 0.77 | [-1.91-1.41] |
| △Triglyceride | -0.15±0.65 | -0.02±1.2 | -0.11 | 0.29 | [-0.37-0.11] |
| △HDL | -0.11±028 | -0.12±0.27 | 0.51 | 0.61 | [-0.05-0.08] |

NLR, neutrophile-to-lymphocyte ratio; SII, systemic immunoinflammatory index; PLR, platelet-to-lymphocyte ratio; LMR, lymphocyte-to-monocyte ratio; PNI, prognostic nutritional index; SIINI is the product of the neutrophil count, platelet count, and hemoglobin level divided by the product of the lymphocyte count, body mass index (BMI), and albumin level; △, the change before neoadjuvant chemotherapy minus the change before operation.

Table 7. Comparison of body composition indicators between patients with and without postoperative complications

| Body composition indicators | Postoperative complication group(n=118） | Non-postoperative-complication group (n=177) | t/z | p | 95% Confidence interval |
| --- | --- | --- | --- | --- | --- |
| SATI | 25.25±19.10 | 24.25±16.93 | 0.47 | 0.64 | [-3.17-5.17] |
| IMATI | 2.49±2.39 | 2.25±1.62 | 1.01 | 0.31 | [-0.22-0.69] |
| VATI | 24.52±17.62 | 25.46±18.83 | -0.43 | 0.67 | [-5.23-3.36] |
| SMI | 41.14±8.39 | 45.14±7.21 | -3.62 | <0.001 | [-5.11--1.51] |
| SMD | 41.62±6.67 | 42.36±6.10 | -0.98 | 0.33 | [-2.22-0.75] |
| IMFD | -66.96±10.94 | -66.65±9.55 | -0.26 | 0.79 | [-2.68-2.06] |
| SFD | -81.39±13.89 | -82.68±12.53 | 0.83 | 0.41 | [-1.78-4.34] |
| VFD | -85.24±10.20 | -85.48±11.37 | 0.19 | 0.85 | [2.31-2.8] |
| SMA | 116.81±27.21 | 128.15±23.09 | -3.84 | <0.001 | [-17.14--5.53] |
| IMFA | 6.94±6.67 | 6.37±4.45 | 0.7 | 0.52 | [-0.81-1.78] |
| SFA | 73.54±48.93 | 74.91±51.97 | -0.23 | 0.82 | [-13.24-10.51] |
| VFA | 65.42±53.06 | 66.49±49.66 | -0.18 | 0.86 | [-13..00-10.87] |
| △SMA | 5.19±9.29 | 4.24±10.81 | 0.78 | 0.43 | [-1.44-3.35] |
| △SFA | 3.85±21.96 | 3.68±20.18 | 0.07 | 0.95 | [-4.72-5.06] |
| △VFA | 1.93±27.78 | 0.76±31.59 | 0.33 | 0.75 | [-5.88-8.21] |
| △IMFA | 7.61±12.35 | 6.56±12.40 | 0.71 | 0.48 | [-1.85-3.94] |
| △SMD | 1.29±3.89 | 0.66±3.96 | 1.36 | 0.18 | [-0.29-1.56] |
| △SAD | -2.63±9.45 | -1.05±9.52 | -1.4 | 0.16 | [-3.8-0.64] |
| △VAD | -2.29±8.8 | -2.92±10.36 | 0.54 | 0.59 | [-1.66-2.91] |
| △IMFD | 1.24±11.68 | 0.72±9.37 | 0.42 | 0.67 | [-1.91-2.94] |
| △SMI | 1.89±3.27 | 1.51±3.83 | 0.87 | 0.39 | [-0.47-1.22] |
| △IMATI | -0.09±1.98 | 0.09±1.34 | -0.9 | 0.37 | [-0.55-0.21] |
| △SATI | 2.95±13.27 | 3.58±13.26 | -0.4 | 0.69 | [-3.74-2.47] |
| △VATI | -0.68±12.46 | -1.91±14.78 | 0.74 | 0.46 | [-2.03-14.78] |

Table 8. Comparison of hematological indicators between the complication group and non-complication group

| Various indicators | Postoperative complication group(n=118) | Non-postoperative-complication group(n=177) | t/z | p | 95% Confidence interval |
| --- | --- | --- | --- | --- | --- |
| NLR | 2.99±4.08 | 2.26±1.43 | 2.2 | 0.03 | [0.078-1.39] |
| SII | 242.72±420.67 | 187.42±188.78 | 1.51 | 0.13 | [-16.67-125.27] |
| PLR | 125.51±126.14 | 107.32±61.45 | 1.65 | 0.1 | [-3.52-39.91] |
| LMR | 5.47±22.29 | 3.84±2.45 | 0.97 | 0.33 | [-1.69-4.96] |
| PNI | 401.31±48.54 | 412.76±40.79 | -2.19 | 0.03 | [-21.75--1.15] |
| SIINI | 65.77±116.25 | 39.26±34.31 | 2.86 | <0.001 | [8.23-44.78] |
| Hemoglobin | 119.28±21.86 | 125.63±22.96 | -2.37 | 0.02 | [-11.61--1.07] |
| Albumin | 43.69±30.59 | 41.28±4.08 | 0.81 | 0.42 | [-3.48-8.32] |
| Platelet | 136.06±67.25 | 133.86±64.66 | 0.28 | 0.78 | [-13.17-17.56] |
| Cholinesterase | 5.5±1.49 | 6.09±5.14 | -1.23 | 0.22 | [-1.55-0.36] |
| Triglyceride | 1.35±0.66 | 1.41±0.68 | -0.79 | 0.43 | [-0.22-0.09] |
| High-density lipoprotein | 1.18±0.33 | 1.21±0.31 | -0.87 | 0.38 | [-0.11-0.04] |
| △NLR | -0.17±4.08 | 0.68±2.38 | -2.26 | 0.03 | [-1.59--0.11] |
| △SII | 158.42±501.33 | 233.12±597.47 | -1.12 | 0.26 | [-205.93-56.53] |
| △PLR | 46.79±139.14 | 72.64±145.48 | -1.52 | 0.13 | [-59.29-7.59] |
| △LMR | 0.57±28.77 | 1.89±16.65 | -0.5 | 0.62 | [-6.54-3.89] |
| △PNI | 19.31±63.53 | 17.62±42.07 | 0.27 | 0.04 | [-10.41-13.78] |
| △SIINI | 19.41±124.32 | 48.85±81.12 | -2.46 | 0.01 | [-52.97--5.9] |
| △Albumin | 1.93±6.38 | 1.76±4.21 | 0.27 | 0.79 | [-1.05-1.38] |
| △Hemoglobin | -5.04±138.72 | 10.49±19.36 | -1.47 | 0.14 | [-36.34-5.26] |
| △Platelet | 87.89±91.17 | 94.86±81.19 | -0.69 | 0.49 | [-27.42-13.47] |
| △Cholinesterase | 1.93±9.04 | 0.59±5.19 | 1.6 | 0.11 | [-0.31-2.97] |
| △Triglyceride | 0.12±1.44 | -0.2±0.59 | 2.66 | 0.008 | [0.08-0.56] |
| △High-density lipoprotein | -0.12±0.31 | -0.11±2.57 | -0.14 | 0.89 | [-0.07-0.06] |

Table 9. Comparative analysis of body composition of patients in various subgroups

| Body composition indicators | Group A (83) | Group B (32) | Group C (83) | Group D (97) | F | P |
| --- | --- | --- | --- | --- | --- | --- |
| SATI | 26.31±18.68 | 28.49±19.56 | 24.87±18.65 | 21.78±15.38 | 1.59 | 0.19 |
| IMATI | 2.28±1.69 | 3.26±3.59 | 2.21±1.72 | 2.23±1.53 | 0.87 | 0.46 |
| VATI | 25.47±17.09 | 31.34±20.93 | 22.81±15.69 | 24.64±20.25 | 1.71 | 0.17 |
| SMI | 45.05±7.44 | 42.91±9.68 | 41.61±8.17 | 44.94±6.87 | 3.62 | 0.015 |
| SMD | 42.24±5.46 | 41.09±9.09 | 41.60±5.56 | 42.62±6.59 | 0.56 | 0.64 |
| IMFD | -67.03±9.86 | -67.57±9.13 | -66.22±11.55 | -66.76±9.40 | 0.17 | 0.92 |
| SFD | -84.08±12.39 | -84.28±15.38 | -80.73±13.27 | -81.06±12.60 | 1.44 | 0.23 |
| VFD | -85.66±13.36 | -87.48±10.49 | -84.67±10.34 | -85.06±9.08 | 0.55 | 0.65 |
| SMA | 128.88±22.67 | 120.76±30.51 | 115.56±26.67 | 127.05±23.09 | 4.58 | 0.005 |
| IMFA | 6.46±4.56 | 9.18±9.91 | 6.09±4.87 | 6.28±5.45 | 0.95 | 0.42 |
| SFA | 76.87±52.03 | 87.76±57.17 | 69.7±43.75 | 71.77±52.69 | 1.13 | 0.34 |
| VFA | 71.17±50.6 | 81.96±60.79 | 62.14±49.32 | 59.79±48.33 | 1.98 | 0.12 |
| △SMA | 3.35±10.92 | 1.72±6.80 | 6.41±10.03 | 5.14±10.50 | 2.23 | 0.09 |
| △SFA | 1.96±18.89 | -3.69±25.67 | 6.18±19.89 | 5.66±21.19 | 2.23 | 0.09 |
| △VFA | -0.45±33.58 | -2.18±33.58 | 1.66±20.76 | 3.43±31.88 | 0.39 | 0.75 |
| △IMFA | 6.31±12.12 | 7.28±13.04 | 8.97±15.22 | 5.76±9.23 | 1.11 | 0.34 |
| △SMD | 0.75±3.58 | 0.78±3.52 | 1.69±3.90 | 0.43±4.35 | 1.63 | 0.18 |
| △SAD | 0.11±9.03 | -1.49±9.55 | -3.09±9.18 | -2.07±10.04 | 1.66 | 0.18 |
| △VAD | -3.3±12.96 | -2.46±9.15 | -2.52±8.08 | -2.32±8.03 | 0.17 | 0.92 |
| △IMFD | 2.09±8.33 | 3.96±15.33 | -0.14±9.59 | -0.115±10.36 | 1.94 | 0.12 |
| △SMI | 1.12±3.78 | 0.64±2.33 | 22.32±3.55 | 1.89±3.78 | 2.57 | 0.055 |
| △IMATI | 0.13±1.49 | -0.84±3.34 | 0.2±0.98 | 0.05±1.21 | 1.17 | 0.32 |
| △SATI | 1.37±11.38 | 1.59±16.8 | 3.06±11.79 | 5.79±14.39 | 1.83 | 0.15 |
| △VATI | -0.78±13.53 | -3.44±15.76 | -0.03±10.66 | -2.49±15.9 | 0.75 | 0.52 |

SATI, subcutaneous fat index; IMATI, intermuscular fat index; VATI, visceral fat index; SMI, skeletal muscle mass index; SMD, skeletal muscle density; IMFD, intermuscular fat density; SFD, subcutaneous fat density; VFD, visceral fat density; SMA, skeletal muscle area; IMFA, intermuscular fat area; SFA, subcutaneous fat area; VFA, visceral fat area; △, the change before neoadjuvant chemotherapy minus the change before operation.

Table 10. Comparative analysis of hematological indicators of patients in various subgroups

| Hematological indicators | Group A (83) | Group B (32) | Group C (83) | Group D (97) | F | P |
| --- | --- | --- | --- | --- | --- | --- |
| SII | 178.54±135.05 | 412.75±729.40 | 180.73±189.11 | 192.47±222.83 | 1.14 | 0.34 |
| PLR | 97.96±53.62 | 141.58±111.60 | 119.24±133.24 | 115.95±66.44 | 2.56 | 0.06 |
| LMR | 3.99±1.57 | 3.34±1.55 | 6.29±26.56 | 3.77±3.04 | 0.63 | 0.59 |
| PNI | 417.89±37.58 | 402.57±53.14 | 397.62±47.56 | 410.76±4.28 | 3.28 | 0.024 |
| Hemoglobin | 125.01±22.30 | 123.82±24.31 | 118.73±21.21 | 124.92±23.56 | 1.44 | 0.23 |
| Albumin | 41.79±3.76 | 40.26±5.31 | 44.83±47.14 | 41.08±25.21 | 0.43 | 0.73 |
| Platelet | 132.34±68.76 | 144.06±72.81 | 131.30±61.51 | 136.67±64.43 | 0.35 | 0.79 |
| Cholinesterase | 5.83±1.10 | 5.70±1.52 | 5.45±1.53 | 6.29±6.87 | 0.65 | 0.59 |
| Triglyceride | 1.40±0.73 | 1.33±0.63 | 1.38±0.67 | 1.39±0.64 | 0.09 | 0.96 |
| High-density lipoprotein | 1.24±0.34 | 1.12±0.31 | 1.19±0.33 | 1.19±0.29 | 1.08 | 0.36 |
| △NLR | 0.56±1.55 | -0.47±4.31 | -0.08±4.06 | 0.78±2.89 | 1.91 | 0.13 |
| △SII | 221.73±529.61 | 87.92±753.93 | 181.68±368.12 | 243.89±647.44 | 0.69 | 0.56 |
| △PLR | 64.64±83.2 | 38.78±122.87 | 45.88±146.06 | 82.09±181.83 | 1.29 | 0.28 |
| △LMR | 3.41±24.05 | 1.03±2.02 | 0.47±34.34 | 0.50±3.26 | 0.33 | 0.81 |
| △PNI | 13.37±40.79 | 25.81±53.86 | 22.41±67.39 | 16.49±43.26 | 0.68 | 0.57 |
| △SIINI | 37.85±43.11 | 21.81±112.87 | 18.19±131.08 | 57.59±101.73 | 2.02 | 0.12 |
| △Albumin | 1.34±4.08 | 2.58±5.39 | 2.24±6.78 | 1.65±4.32 | 0.67 | 0.57 |
| △Hemoglobin | 9.20±20.10 | 15.19±15.64 | -10.97±164.83 | 9.53±19.92 | 1.17 | 0.32 |
| △Platelet | 89.92±75.82 | 89.59±103.28 | 87.05±87.51 | 99.04±85.33 | 0.34 | 0.79 |
| △Cholinesterase | 0.89±1.25 | 1.11±1.27 | 2.35±10.75 | 0.29±6.92 | 1.34 | 0.26 |
| △Triglyceride | -0.17±0.62 | -0.12±0.67 | 0.18±1.66 | -0.19±0.57 | 2.55 | 0.06 |
| △High-density lipoprotein | -0.14±0.28 | -0.03±0.27 | -0.15±0.31 | -0.09±0.24 | 1.72 | 0.16 |

NLR, neutrophile-to-lymphocyte ratio; SII, systemic immunoinflammatory index; PLR, platelet-to-lymphocyte ratio; LMR, lymphocyte-to-monocyte ratio; PNI, prognostic nutritional index; SIINI is the product of the neutrophil count, platelet count, and hemoglobin level divided by the product of the lymphocyte count, body mass index (BMI), and albumin level; △, the change before neoadjuvant chemotherapy minus the change before operation.

Table 11. Correlation analysis between △SFA and hematological nutritional indexes.

| Items | Correlation coefficient | P |
| --- | --- | --- |
| PNI | -0.12 | 0.04 |
| SIINI | -0.046 | 0.43 |
| Albumin | -0.12 | 0.039 |
| Hemoglobin | -0.177 | 0.02 |
| Triglyceride | -0.003 | 0.96 |
| △PNI | 0.12 | 0.048 |
| △SIINI | 0.04 | 0.49 |
| △Albumin | 0.117 | 0.046 |
| △Hemoglobin | 0.24 | 0.068 |
| △Triglyceride | 0.034 | 0.56 |

Table 12. Correlation analysis between △SMI and hematological nutritional indexes.

| Items | Correlation coefficient | P |
| --- | --- | --- |
| PNI | -0.06 | 0.34 |
| SIINI | 0.009 | 0.88 |
| Albumin | -0.56 | 0.34 |
| Hemoglobin | -0.15 | 0.01 |
| Triglyceride | -0.02 | 0.72 |
| △PNI | 0.05 | 0.4 |
| △SIINI | -0.01 | 0.86 |
| △Albumin | 0.05 | 0.4 |
| △Hemoglobin | 0.08 | 0.17 |
| △Triglyceride | 0.097 | 0.095 |

Table 13. Correlation analysis between △SMA and hematological nutritional indexes.

| Items | Correlation coefficient | P |
| --- | --- | --- |
| PNI | -0.05 | 0.395 |
| SIINI | 0.006 | 0.92 |
| Albumin | -0.05 | 0.39 |
| Hemoglobin | -0.14 | 0.016 |
| Triglyceride | -0.02 | 0.71 |
| △PNI | 0.049 | 0.404 |
| △SIINI | -0.011 | 0.85 |
| △Albumin | 0.049 | 0.4 |
| △Hemoglobin | 0.082 | 0.16 |
| △Triglyceride | 0.083 | 0.15 |
